# Expression of Immune-Related Genes of Ducks Infected with Avian Pathogenic *Escherichia coli* (APEC)

**DOI:** 10.3389/fmicb.2016.00637

**Published:** 2016-05-03

**Authors:** Rong Li, Ning Li, Jinzhou Zhang, Yao Wang, Jiyuan Liu, Yumei Cai, Tongjie Chai, Liangmeng Wei

**Affiliations:** ^1^College of Animal Science and Veterinary Medicine, Shandong Agricultural UniversityTai’an, China; ^2^Sino-German Cooperative Research Centre for Zoonosis of Animal Origin Shandong ProvinceTai’an, China; ^3^Collaborative Innovation Centre for the Origin and Control of Emerging Infectious Diseases of Taishan Medical CollegeTai’an, China

**Keywords:** Cherry Valley ducks, APEC, Host immune responses, TLRs, AvBDs, MHCs

## Abstract

Avian pathogenic *Escherichia coli* (APEC) can cause severe disease in ducks, characterized by perihepatitis, pericarditis, and airsacculitis. Although the studies of bacteria isolation and methods of detection have been reported, host immune responses to APEC infection remain unclear. In response, we systemically examined the expression of immune-related genes and bacteria distribution in APEC-infected ducks. Results demonstrated that APEC can quickly replicate in the liver, spleen, and brain, with the highest bacteria content at 2 days post infection. The expression of toll-like receptors (TLRs), avian β-defensins (AvBDs) and major histocompatibility complex (MHC) were tested in the liver, spleen, and brain of infected ducks. TLR2, TLR4, TLR5, and TLR15 showed different expression patterns, which indicated that they all responded to APEC infection. The expression of AvBD2 was upregulated in all tested tissues during the 3 days of testing, whereas the expression of AvBD4, AvBD5, AvBD7, and AvBD9 were downregulated, and though MHC-I was upregulated on all test days, MHC-II was dramatically downregulated. Overall, our results suggest that APEC can replicate in various tissues in a short time, and the activation of host immune responses begins at onset of infection. These findings thus clarify duck immune responses to APEC infection and offer insights into its pathogenesis.

## Introduction

Ducks from the largest waterfowl-breeding industry in China, with a population of up to 20–30 billion per year. Duck morbidity and mortality are more commonly caused by bacterium than viruses, and numerous kinds of infectious bacterial pathogens, including *Pasteurella multocida, Salmonella enterica, Riemerella anatipestifer*, and *Escherichia coli* *(E. coli*), have been reported to threaten duck health throughout the world (Saif et al., 2003; Wei et al., 2013). For one, the pathogen of colibacillosis, avian pathogenic *E. coli* (APEC), can infect ducks of different ages. Actually, APEC induces different syndromes in poultry, such as acute colibacillosis, respiratory colisepticemia, salpingitis, and yolk sac infection (Dho-Moulin and Fairbrother, 1999). For colibacillosis in particular, especially in 4–9 week-old broiler chickens and ducks, the most common symptom is respiratory disease, usually followed by systemic infection with characteristic fibrinous lesions such as airsacculitis, perihepatitis, and pericarditis, as well as fatal septicemia (Guabiraba and Schouler, 2015). At least one study has shown that colibacillosis is typically induced and enhanced by predisposing pathogens, such as viral infections and environmental factors (Ewers et al., 2003).

For hosts, the innate immune response represents the first line of defense against enteric pathogens (Mogensen, 2009). When pathogens invade, the innate immune response manages the invasion by inducing programmed cell death, for example, and secreting pro-inflammatory compounds that direct immune cells to infection sites (Mogensen, 2009; Takeuchi and Akira, 2010). In that context, toll-like receptors (TLRs) serve as important pattern recognition receptors (PRRs) that can recognize various pathogenic organisms, to recognize a variety of pathogenic motifs, including those of peptidoglycan, lipoproteins, lipopolysaccharide, double-stranded viral RNA, and unmethylated bacterial CpG DNA ( Keestra et al., 2013). Upon their discovery, TLRs were shown to function as essential receptors for embryonic development and antifungal response in *Drosophila* (Lemaitre et al., 1996). Once TLRs recognize the corresponding ligands, transcription factors become activated, thereby inducing not only the expression of interferon-stimulated genes and pro-inflammatory cytokines, but also the formation of innate immune response to causative agents (Kawai and Akira, 2010; Chen et al., 2013). Studies to date have reported that five duck (*Anas platyrhynchos*) TLRs (duTLRs): duTLR2 (Huang et al., 2011), duTLR3 (Jiao et al., 2012), duTLR4 (Jia et al., 2012), duTLR5 (Xiong et al., 2014), and duTLR7 (MacDonald et al., 2008), and all are involved in recognizing the different molecular patterns of microorganisms and their own components.

In a variety of organisms, host defense peptides consist of small cationic peptides usually with only 30–45 amino acids, often positively charged ones. Defensins play an essential role against pathogens, including bacteria, fungi, and certain enveloped viruses (Harwig et al., 1994; Evans et al., 1995; Froy and Gurevitz, 2003; Hiemstra, 2007). They moreover represent the immunomodulatory properties in T lymphocytes (Chertov et al., 1996), monocytes (Territo et al., 1989), immature dendritic cells (Yang et al., 1999), and mast cells (Niyonsaba et al., 2002). According to the disulfide-bonding pattern, defensins can be subdivided into three subfamilies, namely α-, β-, and 𝜃-defensins. All three defensin subfamilies have been demonstrated in mammals and humans, though only β-defensins were found in birds (Sugiarto and Yu, 2004). At present, all avian β-defensins have been assigned gene names as avian β-defensins (AvBDs). Since the first AvBD’s discovery in the mid-1990s (Evans et al., 1994), more than 40 known isoforms of AvBDs have been identified in birds, including chickens, ducks, geese, and quail (Lynn et al., 2004, 2007; Ma et al., 2008, 2009a,b, 2011, 2012a,c; Wang et al., 2010). Defensins execute anti-microbial activity by non-oxidative mechanisms (Sahl et al., 2005) and some act as chemoattractants for lymphocytes, monocytes and dendritic cells as well (Yang et al., 1999; Ganz, 2003).

Major histocompatibility complex (MHC) is a highly polymorphic gene group. MHC class I (MHC-I) molecules’ proteins are critical for immune defenses against pathogens; as ligands for CD8^+^ T cells, they activate cytotoxic lymphocytes (CTL) and the subsequent lysis of target cells (Bjorkman and Parham, 1990; Garboczi et al., 1996). MHC-I also serve as self-recognition elements for natural killer cells. By contrast, MHC class II (MHC-II) molecules present antigenic peptides on CD4^+^ T cells and are critical for initiating the adaptive immune response. In chickens, MHCs are primarily divided into MHC-I, MHC-II, and MHC-IV, whereas the studies on the MHCs of ducks are relatively less compare to the chicken (Zhang et al., 2010). Other than their roles innate immune response, MHCs also have an important function in humoral immunity and cellular immunity, as well as act as a bridge between innate and adaptive immune responses.

To study the immune responses of ducks infected with APEC, we used *E. coli O1:K1* to infect 4-week-old Cherry Valley ducks. We systematically investigated the expression of TLRs, AvBDs and MHCs in the liver, spleen, and brain, as well as the bacteria content in those tissues, at 1, 2, and 3 days post infection (dpi). Ultimately, our findings clarify the immune responses of ducks infected with APEC.

## Materials and Methods

### Bacterial Strains and Animals

The APEC (*O1:K1*) strain used was isolated from clinically infected ducks suffering from colibacillosis and housed in the Environmental Microbiology Laboratory at Shandong Agricultural University. The bacterial strain was cultivated in Luria-Bertani (LB) agar at 37°C for 18 h, after which a single colony was inoculated in 5 mL of LB broth and cultivated at 37°C for 18 h with agitation. Healthy, 1-day-old healthy Cherry Valley ducks were purchased from a duck farm (Tai’an, Shandong, China) and housed in isolators until used.

### Animal Experiments

Twenty-eight 4-week-old ducks were randomly divided into two groups of 14. The experimental group was subcutaneously inoculated in the neck with 0.3 mL of an overnight culture in LB-Miller broth the inoculum in the stationary phase was 10^9^ colony-forming units (CFU) as previously described (Dho and Lafont, 1984). The control group was inoculated with 0.3 mL LB-Miller broth in the same manner. At 1, 2, and 3 days post infection (dpi), three live ducks from each group were euthanized and parallel tissues of the liver, spleen, and brain were collected and stored at −70°C to isolate the APEC strain and the analyze immune-related gene expression.

Bacteria were re-isolated from the liver, spleen, and brain, beginning by weighing the tissue samples and suspending them in phosphate-buffered saline (1 mL/g). Serial 10-fold dilutions were plated onto LB agar and incubated at 37°C for 24 h, after which, colonies were counted to determine the CFU per gram in each organ. The rest of the ducks were observed for clinical symptoms until 14 dpi. All animal experiments were performed according to the guidelines of the Committee on the Ethics of Animals of Shandong and the appropriate biosecurity guidelines, and the protocol was approved by Shandong Agricultural University Animal Care and Use Committee (No. SDAUA-2015-004).

### RNA Isolation and cDNA Synthesis

Total RNA was extracted from the samples of Cherry Valley ducks using TRIzol reagent (Takara, Dalian, China) according to the manufacturer’s instructions. Total RNA (1 μg) was reverse transcribed using the SuperScript III First Strand synthesis kit (Life Technologies, Carlsbad, CA, USA), and synthesized cDNA was stored at −20°C.

### Quantitative Real-Time Polymerase Chain Reaction

Quantitative real-time polymerase chain reaction (qRT-PCR) was performed with the QuantiFast SYBR Green PCR kit (Qiagen, Hilden, Germany). Some primers used for qRT-PCR were designed with Primer3^1^ based on published sequences, whereas others were designed as previously reported (Adams et al., 2009; Ma et al., 2011, 2012b). Primer pairs (**Table 1**) were selected based on specificity determined by dissociation curves, and qRT-PCR was performed using a 7500 Fast Real-Time PCR system (Applied Biosystems, Carlsbad, CA, USA). PCR entailed one cycle of 95°C for 30 s, followed by 40 cycles of 95°C for 5 s and 60°C for 34 s. Dissociation curves of the products were identified at the final step of the PCR. For the purposes of assay validation, purified products were cloned into pMD19-T and sequenced to verify correct target amplification.

**Table 1:** Primers used in this study.

Primer name	Sequence(5′–3′)	Product size (bp)	GenBank no.
TLR2 F	AAGAAAATGGAGCTGCTGGA	231	HQ166194.1
TLR2 R	GAAAAACACAGCGCAGATCA		
TLR4 F	ACCCATTGTCACCAACATCATC	195	JN048668.1
TLR4 R	TGCCTCAGCAAGGTCTTATTCA		
TLR5 F	GAACTCCGGCTGTTTCACAACA	199	KF316966.1
TLR5 R	TGCTTTCACACAGTTTGGATATGTC		
TLR15 F	AGAAGCACAAGCTCCCAAAA	152	JN618074.1
TLR15 R	CAAATGTGCCAGGTTCAATG		
AvBD2 F	TCCAGGTTTCTCCAGGATTG	93	FJ465147.1
AvBD2 R	TCAGGTGGATGGGACATCTT		
AvBD4 F	ATCGTGCTCCTCTTTGTGGCAGTTCA	153	–
AvBD4 R	CTACAACCATCTACAGCAAGAATACT		
AvBD5 F	GCTGTCCCTTGCTCGAGGATT	139	JF949720.1
AvBD5 R	GGAATACCATCGGCTCCGGC		
AvBD7 F	GGATCCTTTACCTGCTGCTG	129	JF831960.1
AvBD7 R	TTCGACAGATCCCTGGAAAG		
AvBD9 F	ATGAGAATCCTTTTCTTCCTTGTTGC	204	EF431957
AvBD9 R	TTAGGAGCTAGGTGCCCATTTGCAGC		
AvBD16 F	CGCTGCAGGAAACTCTGTC	96	JQ359445.1
AvBD16 R	CCCGAACATCTCCCAATATG		
MHC-I F	GAAGGAAGAGACTTCATTGCCTTGG	196	AB115246
MHC-I R	CTCTCCTCTCCAGTACGTCCTTCC		
MHC-II F	CCACCTTTACCAGCTTCGAG	229	AY905539
MHC-II R	CCGTTCTTCATCCAGGTGAT		
β-actin F	GGTATCGGCAGCAGTCTTA	160	EF667345.1
β-actin R	TTCACAGAGGCGAGTAACTT		

### Statistical Analysis

All data were expressed as fold change in gene expression and calculated using the 2^−ΔΔCt^ method. Housekeeping gene β-actin was used as an endogenous control to normalize the expression level of target genes and logarithmic transformation was applied to all fold change values. Data were analyzed with a Student’s *t*-test using GraphPad Prism 5 (GraphPad Software Inc., San Diego, CA, USA). Statistical significance was set at *P* < 0.05.

## Results

### Clinical Signs and Gross Lesions of APEC-infected Ducks

Throughout the experiment, the control group exhibited no unusual clinical signs. By contrast, APEC-infected ducks demonstrated listlessness, ruﬄed feathers, anorexia, inactivity, and dyspnea as early as 1 dpi. At 2 dpi, the clinical signs became more severe, and there ducks died; another duck died at 3 dpi and one more at 4 dpi.

In addition, APEC-infected ducks also showed gross lesions, including typical fibrinous pericarditis, liver surface layers with yellowish-white fibrinous exudates, peritoneal adhesions, swollen, and cloudy airsac with yellow fibrinous exudates, kidney swelling and more yellowish-white fibrinous exudates in the abdominal cavity and intestinal surface (data not shown). However, no clear pathological changes were observed in the various organs and tissues in the control group.

### Bacteria Content in APEC-infected Ducks

**FIGURE 1:**
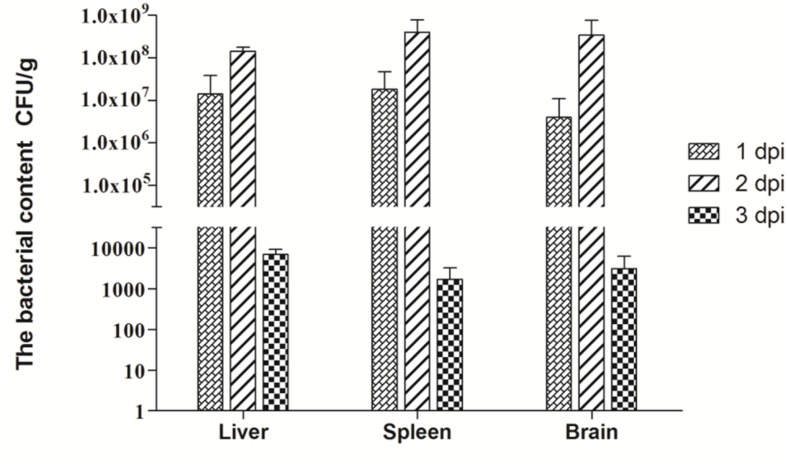
**Bacteria content of avian pathogenic ***Escherichia coli*** (APEC)-infected ducks at 1, 2, and 3 dpi.** Data were expressed as means ± standard deviations (*n* = 3) and each sample was analyzed in triplicate.

We also tested the bacteria content in the liver, spleen and brain at 1, 2, and 3 dpi. As **Figure 1** shows, at 1 dpi, APEC replicated rapidly in all tested tissues. In the liver, bacteria content reached 1.4 × 10^7^ CFU/g, while those in the spleen and brain reached 1.8 × 10^7^ and 4 × 10^6^ CFU/g, respectively. At 2 dpi, the bacteria content peaked in the spleen, liver, and brain tissues at 4 × 10^8^, 1.4 × 10^8^, and 3.4 × 10^8^ CFU/g, respectively. However, bacteria contents in all tissues tested dramatically declined from 2 to 3 dpi and reached only 10^3^ CFU/g. On the whole, APEC could replicate quickly in multiple organs, thereby causing systemic impairment.

### Expression of TLR mRNA in APEC-infected Ducks

To determine the expression of TLRs in host immune responses to APEC infection, we studied the production levels of TLR2, TLR4, TLR5, and TLR15 in the liver, spleen, and brain of ducks at 1, 2, and 3 dpi.

**FIGURE 2:**
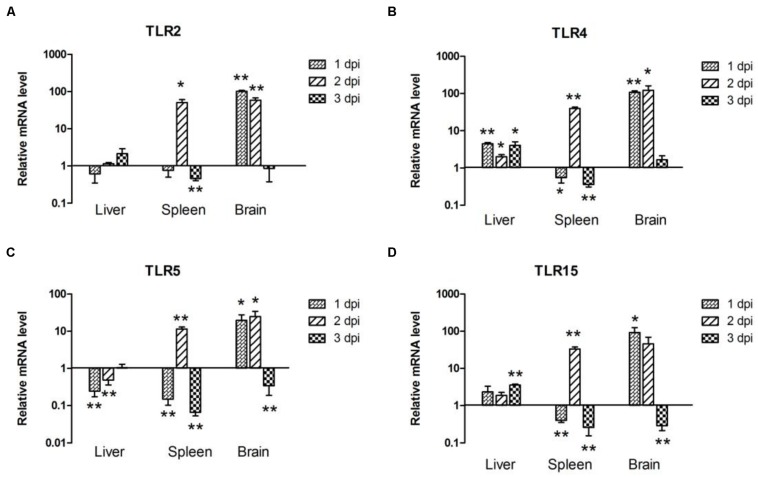
**The expression of toll-like receptors (TLRs) in the liver, spleen and brain from the APEC-infected ducks. (A)** TLR2, **(B)** TLR4, **(C)** TLR5, **(D)** TLR15. *Y*-axis represents the fold change of target genes in the experiment groups versus those in control group. Data were expressed as means ± standard deviations (*n* = 3). The difference was calculated by the Student’s *t*-test. ^∗^*P* < 0.05, ^∗∗^*P* < 0.01.

In the liver, the expression of TLR2 was downregulated by 0.62-fold at 1 dpi (*P* > 0.05), then upregulated by 1.14-fold (*P* > 0.05), and 2.14-fold (*P* > 0.05) at 2 and 3 dpi, respectively (**Figure 2A**). TLR4 and TLR15 expression was upregulated throughout the 3 days of testing, and a significant difference occurred at 3 dpi (*P* < 0.05; **Figures 2B,D**). By contrast, TLR5 mRNA expression was downregulated at 1 and 2 dpi, followed by upregulation at 3 dpi (1.54-fold, *P* > 0.05; **Figure 2C**). In the spleen, the expression of TLR2, TLR4, TLR5, and TLR15 followed a similar trend; downregulation at 1 and 3 dpi, followed by marked upregulation at 2 dpi (50.68-fold, *P* < 0.05; 39.82-fold, *P* < 0.01; 11.51-fold, *P* < 0.01 and 33.41-fold, *P* < 0.01, respectively; **Figure 2**). In the brain, TLR2 and TLR15 mRNA expression was induced at 1 dpi by 101.60-fold (*P* < 0.01; **Figure 2A**) and 92.24-fold (*P* < 0.05; **Figure 2D**), respectively, decreased slightly at 2 dpi by 58.48-fold (*P* < 0.01; **Figure 2A**) and 46.05-fold (*P* > 0.05; **Figure 2D**), and suppressed at 3 dpi by 0.86-fold (*P* > 0.05; **Figure 2A**) and 0.28-fold (*P* < 0.01; **Figure 2D**). The expression of TLR4 and TLR5 was upregulated at 1 dpi (108.80-fold, *P* < 0.01 and 19.79-fold, *P* < 0.05, respectively) and gradually increased at 2 dpi (122.60-fold, *P* < 0.05 and 24.72-fold, *P* < 0.05, respectively; **Figures 2B,C**). As with the TLR2 and TLR15, there was also a decrease in TLR4 and TLR5 expression at 3 dpi, by 1.65-fold (*P* > 0.05; **Figure 2B**) and 0.34-fold (*P* < 0.01; **Figure 2C**), respectively. Taken together, TLRs such as TLR2 and TLR4 were involved in the host immune response to APEC infection.

### Expression of AvBDs mRNA in APEC-infected Ducks

**FIGURE 3:**
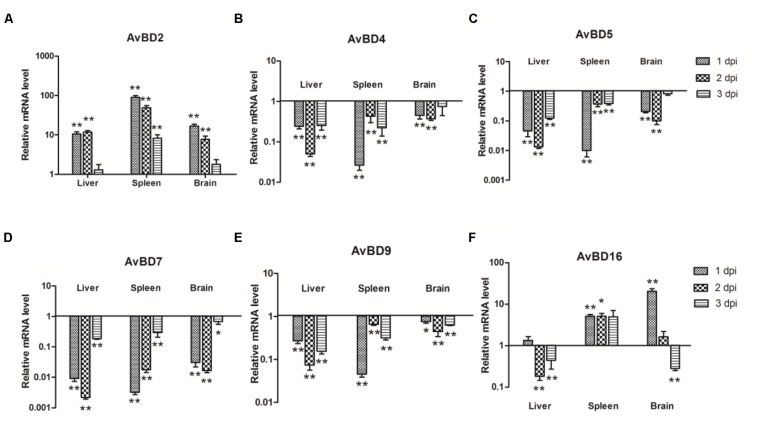
**The expression of avian β-defensins (AvBDs) in the liver, spleen, and brain from the APEC-infected ducks. (A)** AvBD2, **(B)** AvBD4, **(C)** AvBD5, **(D)** AvBD7, **(E)** AvBD9, **(F)** AvBD16. *Y*-axis represents the fold change of target genes in the experiment groups versus those in control group. Data were expressed as means ± standard deviations (*n* = 3). The difference was calculated by the Student’s *t*-test. ^∗^*P* < 0.05, ^∗∗^*P* < 0.01.

Since AvBDs are effective components of the response to bacterial infections, AvBDs (AvBD2, AvBD4, AvBD5, AvBD7, AvBD9, and AvBD16) expression in the liver, spleen, and brain of ducks was investigated at the early stage of bacterial infection. In all three tissues, the expression of AvBD2 was upregulated throughout testing (**Figure 3A**). In the liver, AvBD2 was induced at 1 dpi (10.48-fold, *P* < 0.01), peaked at 2 dpi with 11.75-fold increase (*P* < 0.01), and decreased at 3 dpi (1.29-fold, *P* > 0.05; **Figure 3A**). AvBD16 mRNA was upregulated by 1.35-fold (*P* > 0.05), but dramatically downregulated at the following 2 days (**Figure 3F**). In the spleen, there was an 89.78-fold increase in the AvBD2 mRNA level at 1 dpi (*P* < 0.01), followed by a gradual decline at 2 dpi (49.13-fold, *P* < 0.01), with an exception in the expression level at 3 dpi (8.22-fold, *P* < 0.01; **Figure 3A**). The expression of AvBD16 occurred at a similar level and had showed significant difference with the control group during testing (*P* < 0.05; **Figure 3F**). In the brain, AvBD2 mRNA expression was significantly upregulated at 1 dpi (16.82-fold, *P* < 0.01; **Figure 3A**), yet gradually dropped following 2 days (7.71-fold, *P* < 0.01 and 1.78-fold, *P* > 0.05, respectively; **Figure 3A**). AvBD16 mRNA expression increased constantly, peaked at 1 dpi in the brain (20.44-fold, *P* < 0.01; **Figure 3F**), yet decreased to 0.28-fold at 3 dpi (*P* < 0.01; **Figure 3F**). However, the expression of AvBD4, AvBD5, AvBD7, and AvBD9 was downregulated in the tested tissues during the first 3 days (**Figures 3B–E**). These data suggest that APEC infection can downregulate most AvBDs in ducks, though the expression of AvBD2 and AvBD16 was upregulated, which indicates their potentially pivotal role in defense against pathogens.

### Expression of MHC-I and -II Molecules in APEC-infected Ducks

**FIGURE 4:**
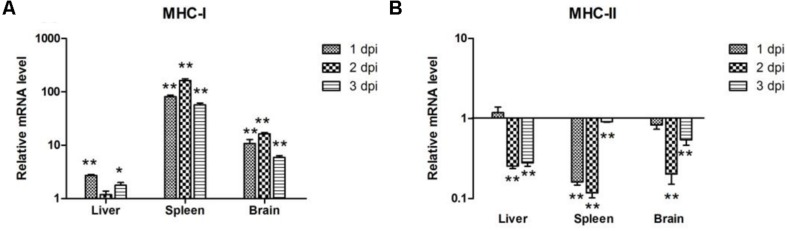
**The expression of MHC-I and -II in the liver, spleen, and brain from the APEC-infected ducks. (A)** MHC-I, **(B)** MHC-II. *Y-*axis represents the fold change of target genes in the experiment groups versus those in control group. Data were expressed as means ± standard deviations (*n* = 3). The difference was calculated by the Student’s *t*-test. ^∗^*P* < 0.05, ^∗∗^*P* < 0.01.

To determine the induction of MHC-I and -II molecules in host immune responses, we examined their expression levels at the first 3 days after bacterial infection. MHC-I expression was upregulated in the liver, spleen, and brain of the infected ducks (**Figure 4A**) and showed a similar pattern in the spleen and brain, with the highest value at 2 dpi by 163.17 and 16.23-fold, respectively (*P* < 0.01; **Figure 4A**). In the liver, the expression of MHC-I peaked at 1 dpi by 2.69-fold (*P* < 0.01), then decreased slightly during the next 2 days (**Figure 4A**). However, the expression of MHC-II was downregulated at almost all time points (**Figure 4B**). These results showed that both MHC-I and -II molecules were modulated by APEC.

## Discussion

Pathogenicity in hosts correlates with the pathogen contents in their tissues (Cunnington, 2015). Our results show that APEC can quickly replicate, even up to 10^6^–10^7^ CFU/g at 1 dpi (**Figure 1**). The bacteria content of all tested tissues peaked at 2 dpi with 10^8^ CFU/g and rapidly decreased at 3 dpi (**Figure 1**). Notably, bacterial content in the brain reached the 4 × 10^6^ CFU/g and 3.4 × 10^8^ CFU/g at 1 and 2 dpi, respectively (**Figure 1**). Several studies have demonstrated that *E. coli* can invade brain microvascular endothelial cells and break the blood–brain barrier via its virulence factor (Badger et al., 2000; Wang and Kim, 2002), In our study, APEC was also detected in the brain and replicated rapidly in parenchymal organs, including the brain, thereby indicating that it can generate bacteremia and break the blood–brain barrier after only a brief period. The rapid replication of bacteria caused extensive tissue damage and dysfunction, as consistent with the clinical symptoms and gross lesions of APEC-infected ducks at the onset of the infection.

Avian TLRs are differ slightly from their mammalian counterparts (**Table 2**). For one, in chickens, the TLR2 has duplicated genes chTLR2a and chTLR2b. Previous studies have also shown that TLR4 plays a significant role in the susceptibility of mammals and chickens to systemic salmonellosis (Leveque et al., 2003). Also in mammals and chicken, TLR5 plays an important role in host defense against bacterial infections (Fumitaka Hayashi, 2001). Avian TLR15 is a potential sensor for the recognizing invading viruses and bacteria, and, in at least one study chTLR15 was involved in the immune response against bacterial infections (Higgs et al., 2006). In our study, the expression of all tested TLRs showed significant upregulation in the brain at 1 and 2 dpi and in the spleen at 2 dpi (**Figure 2**). In the brain, all tested TLRs’ expression peaked at 1 or 2 dpi and downregulated at 3 dpi (**Figure 2**); in the spleen, however, all tested TLRs’ expression upregulated and peaked at 2 dpi, but downregulated at 1 and 3 dpi (**Figure 2**). Only slight changes were observable in the tested TLRs in the liver compared to those in the spleen and brain (**Figure 2**). There were nevertheless different expression patterns of TLRs in different tissues, perhaps most likely because different organs were differently sensitive to the bacterial infection; the brain’s sensitivity was strongest, followed by the spleen’s, lastly, the liver’s, which prompted different immune responses to APEC infection in various tissues. Yet, the specific mechanism of that phenomenon requires additional study.

**Table 2:** Comparison of the toll-like receptors (TLR) between human and birds.

Pattern recognition receptor (PRR)	Human	Origin of ligand	Chicken	Duck	Origin of ligand
Membrane-bound PRR (TLR) On plasma membrane	TLR2	Bacteria, fungus;Parasites, virus	TLR2a TLR2b	Present	UnknownBacteria
	TLR4	Bacteria, fungus;Parasites, virus	Present	Present	Unknown
	TLR5	Bacteria	Present	Present	Bacteria
			TLR15	Present	Bacteria; Virus

In sum, the kinetics of bacterial loads and the TLRs’ mRNA expression from the tested tissues were essentially consistent throughout our experiment. The bacteria loads peaked the peak at 2 dpi (**Figure 1**), and the expression of TLRs also reached its maximum (**Figure 2**). The expression of TLRs in all tested tissues was downregulated accompanying the reduction of bacterial loads at 3 dpi (**Figures 1** and **2**), a phenomenon confirming the theory that innate immune response serves as a first line of defense against invading pathogens only has a rapid action time.

To date, several AvBDs have been identified in ducks such as *A. platyrhynchos* AvBD (Apl-AvBD) 1–7, 9, 10, 12, and 16 (Ma et al., 2012b). At the same time, antiviral activity against duck hepatitis virus has been observed in Apl-AvBD4, 7 and 12 (Lemaitre et al., 1996; Takeuchi and Akira, 2009; Chen et al., 2013). Previous studies have furthermore shown that Apl-AvBD1, 3, 5, 6, and 16 were less effective against *E. coli* (MIC = 125 μg/mL; Ma et al., 2012b), and that Apl-AvBD2 inhibited the growth of *E. coli* at a concentration of 25 μg/mL (Soman et al., 2009). However, *Meleagris gallopavo* AvBD2, which exhibits high amino acid similarity with Apl-AvBD2, did not kill *E. coli* (Evans et al., 1994), and duck AvBD9 even exhibited low activity against *E. coli* (Ma et al., 2009a). In agreement with previous studies, we showed that Cherry Valley duck AvBD2 and 16 exhibited strong bactericidal activities against *E. coli* (**Figures 3A,F**), though Cherry Valley duck AvBD4, 5, 7, and 9 demonstrated low activity against *E. coli* (**Figures 3B–E**). Considering the large number of APEC in the various tissues that caused extensive tissue damage and weakened host immune response to APEC infection, infected ducks could not produce more antimicrobial peptides and effectively eliminate bacterias which could have been partly responsible for the death of the ducks.

In ducks, part of the MHC-I region and corresponding cDNA sequences, as well as a complete MHC-II α gene, have been cloned (Xia et al., 2004; Ren et al., 2011). In our experiment, the high expression of MHC-I was detectable in the spleen at 2 dpi (163.17-fold, *P* < 0.01), and MHC-I mRNA expression was almost all significantly upregulated in the three tissues (*P* < 0.05; **Figure 4A**). By contrast, the expression of MHC-II in the tissues showed significant downregulation (*P* < 0.01; **Figure 4B**), which indicated that MHC-I and MHC-II were associated with the immune response in ducks following APEC infection. Though MHC-I is a kind of antigen gene that exists on the surface of all cells, MHC-II presents only on the surface of a few cells (Fooksman, 2014). We accordingly suspected that the expression of the two MHCs related to the different distribution in the cells.

Actually, there exist the interaction between pathogen and host during the process of APEC infected duck. On one hand, the host could resist the infection of APEC, and on the other hand, APEC could invade the body and escape the suppression from the host immunity. For instance, the APEC T6SS2 component organelle trafficking protein (DotU) could elicit antibodies in infected ducks. Deletion of the dotU gene of APEC abolished hemolysin-coregulated protein 1 secretion, leading to the decreased expression of interleukin (IL)-6 and IL-8 genes in HD-11 chicken macrophages (Wang et al., 2014). Another study has shown that *E. coli* type three effectors can manipulate PAMP/MAMP-triggered immune signaling components and acting on the evolutionary conserved cellular hubs of immune responses (Fraiture and Brunner, 2015). The interactions between APEC and host will be a further study in our next research.

Altogether, we have shown that the immune-related genes expression patterns of ducks infected with APEC. Our study suggested that APEC could rapidly replicate in the tested tissues in a short time, and the activation of host immune responses began at early time of infection. Given the roles AvBDs played in the response to bacteria, the downregulated of most AvBDs might be the part reason why ducks were died after APEC infection, but the specific reasons are still investigated in the further study. These results illuminate the immune responses of ducks infected with APEC and offer insights into the pathogenesis of APEC.

## Author Contributions

RL and NL designed and conducted the study, performed most of the experiments, and wrote the manuscript. JZ performed the calculation, and YW and JL collected samples, prepared RNA, and cloned the gp85 gene. RL and NL performed the biological experiments. YC, TC, and LW discussed the results and LW designed the study.

## Conflict of Interest Statement

The authors declare that the research was conducted in the absence of any commercial or financial relationships that could be construed as a potential conflict of interest.

## Footnotes

1 http://bioinfo.ut.ee/primer3-0.4.0/
